# Robert Koch: From Anthrax to Tuberculosis – A Journey in Medical Science

**DOI:** 10.7759/cureus.72955

**Published:** 2024-11-04

**Authors:** Kasireddy Sravanthi, Krishna Sailaja Sattiraju, Sheuli Paul, N G Nihal, Shradha Salunkhe, Shailaja V Mane

**Affiliations:** 1 Paediatrics, Dr. D. Y. Patil Medical College, Hospital and Research Centre, Dr. D.Y. Patil Vidyapeeth (Deemed to be University), Pune, IND; 2 Obstetrics and Gynaecology, Dr. D. Y. Patil Medical College, Hospital and Research Centre, Dr. D.Y. Patil Vidyapeeth (Deemed to be University), Pune, IND; 3 Psychiatry, Gayatri Vidya Parishad Institute of Health Care and Medical Technology, Visakhapatnam, IND

**Keywords:** anthrax, bacteriology, cholera, historical vignette, infectious diseases, koch's postulates, microbiology, nobel prize, robert koch, tuberculosis

## Abstract

The pioneering German physician and microbiologist Heinrich Hermann Robert Koch (1843-1910) made pivotal contributions to the field of bacteriology, significantly advancing the germ theory of disease. His groundbreaking research in identifying the causative agents of anthrax, tuberculosis, and cholera revolutionized medical science and public health. Koch's development of essential microbiological techniques, such as using agar for bacterial cultures and introducing the Petri dish, transformed laboratory practices. Additionally, his formulation of Koch's postulates established a systematic method for linking specific pathogens to diseases, a framework that remains influential today. Koch's remarkable achievements were recognized with numerous prestigious honors, including the Nobel Prize in Physiology or Medicine in 1905. His legacy lives on through institutions like the Robert Koch Institute and World Tuberculosis Day, commemorating his profound impact on global health and infectious disease research. Koch's work continues to serve as a cornerstone in studying and controlling infectious diseases.

## Introduction and background

**Figure 1:**
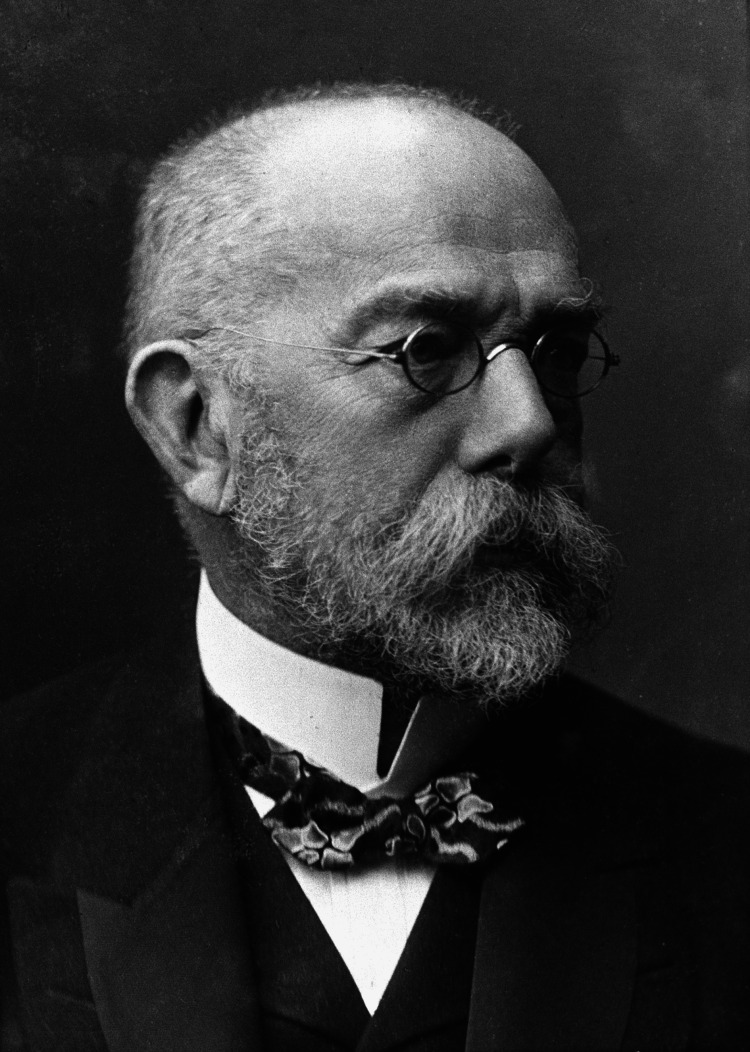
Robert Koch Source: Wikimedia Commons/Public Domain

Heinrich Hermann Robert Koch, a preeminent German physician and microbiologist of the late 19th and early 20th centuries, made substantial contributions to establishing bacteriology as a formal scientific discipline (Figure 1). He made groundbreaking discoveries, identifying the causative bacteria behind tuberculosis, cholera, and anthrax, which significantly advanced the germ theory of disease and had profound implications for public health. Koch's innovative contributions, including the development of techniques such as the oil immersion lens, agar-based bacterial culture methods, and microphotography, revolutionized the field of microbiology. Moreover, his formulation of the renowned Koch's postulates remains a fundamental framework for linking specific microorganisms to their corresponding diseases. Recognized for his remarkable work, Koch was awarded the Nobel Prize in 1905 for his research on tuberculosis. March 24, the date he identified the tuberculosis bacterium, is commemorated as World Tuberculosis Day, honoring his enduring legacy in the medical sciences [1].

## Review

Early life and education

**Figure 2:**
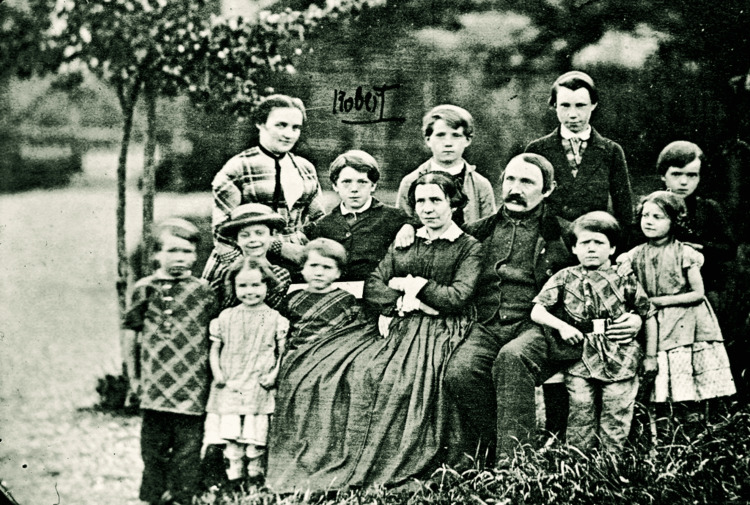
Robert Koch (top row, left next to the mother) with his family in Clausthal in the Harz region (1854) Source: Robert Koch Institute, Berlin Permission obtained from Robert Koch Institute.

Robert Koch was born on December 11, 1843, in Clausthal, Germany. He demonstrated remarkable academic prowess from a young age (Figure 2). As the third of thirteen children, he taught himself to read and write before starting school in 1848 and excelled in science and mathematics throughout his secondary education [2].

Driven by the desire to become a physician, the 19-year-old Koch began his studies in natural sciences at the University of Göttingen. There, he concentrated on mathematics, physics, and botany while also serving as an assistant in the Pathological Museum. After just three semesters, he decided to pursue a career in medicine. In his fifth semester, Koch participated in groundbreaking research on uterine nerve structure under the renowned Jacob Henle, a venture that earned him a prestigious research prize and a brief opportunity to learn from the eminent German physician Rudolf Virchow. During his sixth semester, Koch's research on succinic acid at the Physiological Institute formed the basis of his dissertation. Graduating from medical school in January 1866 with the highest distinction, maxima cum laude, Koch's early academic accomplishments foreshadowed his profound impact on the field of medicine [3,4].

Contributions

Anthrax

Robert Koch is renowned for identifying Bacillus anthracis as the causative agent of anthrax, a deadly disease affecting humans and livestock. As a district physician in Wollstein (now Wolsztyn), Poland, starting in 1872, Koch investigated the disease, which had been causing widespread fatalities without a clear cause. By 1876, Koch discovered that anthrax was caused by a single pathogen and uncovered the role of dormant spores, which could survive in harsh conditions and activate under favorable ones. His groundbreaking work, conducted in a minimally equipped lab, demonstrated that a specific microorganism was responsible for the disease and supported the germ theory of disease. Koch published his findings in 1876 in "Die Ätiologie der Milzbrand-Krankheit" (The Etiology of Anthrax Disease, Based on the Developmental History of Bacillus Anthracis) and later provided the first photographs of bacteria. His method of using stained bacterial cultures on glass slides and observing them under a microscope was crucial because he was the first to establish a direct link between a specific microorganism and a particular disease. This work refuted the concept of spontaneous generation and reinforced the germ theory of disease [5].

Techniques in Bacterial Study

Robert Koch pioneered key advancements in microscopy, including using an oil immersion lens and condenser that enhanced the visibility of smaller microorganisms. He also introduced the innovative technique of microphotography for observing these microbial subjects. Additionally, Koch developed foundational staining methods employing dyes like methylene blue and Bismarck brown to visualize bacterial specimens better. In his quest to cultivate pure bacterial cultures, Koch initially experimented with potato slices, which proved inadequate for many microorganisms. He then turned to nutrient solutions containing gelatin but found that the gelatin melted at 37°C and was degraded by numerous bacteria. In 1881, following a suggestion from his assistants Walther and Fanny Hesse, Koch adopted agar as a culture medium. Agar's unique properties, including its solid state at 37°C, resistance to bacterial degradation, and transparency, rendered it an optimal substrate for cultivating and isolating pure bacterial colonies [6].

Development of Petri Dish

In 1881, Robert Koch published a seminal work titled "Zur Untersuchung von Pathogenen Organismen" (Methods for the Study of Pathogenic Organisms), which became known as the "Bible of Bacteriology." In this publication, Koch introduced a groundbreaking technique for cultivating bacteria. He developed a method using a glass slide coated with a layer of agar, a solidifying agent. Koch employed a novel cultivation method involving the pouring of liquid agar onto a glass slide, followed by a thin gelatin overlay to solidify the medium. The entire assembly was then enclosed within a circular glass chamber, termed a "feuchte Kammer" or "moist chamber." This controlled environment facilitated uniform bacterial distribution and unobstructed observation of microbial growth [7]. Louis Pasteur praised Koch for his innovative plating method when he presented it at the 7th International Medical Congress in London in 1881. This method, employing agar as a solid medium, facilitated the isolation of pure bacterial cultures and enabled significant advancements. Koch's students, including Loeffler and Gaffky, made pivotal discoveries utilizing this approach. In 1887, Petri refined the technique by introducing a circular glass dish as the culture container, minimizing contamination and establishing the ubiquitous Petri dish as a cornerstone of microbiology [7].

Cholera

In August 1883, the German government tasked Robert Koch and a medical team with investigating a cholera outbreak in Alexandria, Egypt. Although Koch observed bacterial infections in the intestines of deceased cholera patients, he initially could not confirm whether these bacteria were the cause of the disease. Following the abatement of the Egyptian epidemic, Koch was reassigned to Calcutta, India, where he confronted a severe outbreak associated with the Ganges River. Through examining nearly 100 autopsies and isolating the same bacteria from water tanks, Koch identified the source of the infection. On January 7, 1884, he isolated the bacterium in pure culture and noted its distinctive comma shape. Koch also discovered that the bacterium could destroy red blood cells and hypothesized that it produced a toxin to cause the disease [8]. This cholera toxin was later identified by Sambhu Nath De in 1959 [9]. Koch communicated his research findings to the German Secretary of State for the Interior in February, subsequently publishing the results in the Deutsche Medizinische Wochenschrift the following month [10]. Although Vibrio cholerae was isolated from patients with cholera at the time of Koch, it was also isolated from healthy subjects, thereby defying the specificity of association demanded by Koch's second postulate [11]. Previously in 1854, a cholera outbreak in London led John Snow to identify the transmission mode as "waterborne" [12]. The bacterium responsible for cholera had been previously documented by Italian physician Filippo Pacini in 1854 and observed by Catalan physician Joaquim Balcells i Pascual, neither scientist definitively established its causal role in the disease. Koch's colleague, Richard Pfeiffer, later confirmed the bacterium as Pacini's vibrioni and renamed it Vibrio cholerae in 1896 [8].

Understanding Tuberculosis: Koch’s Research, Treatment Trials, and the Introduction of Tuberculin

**Figure 3:**
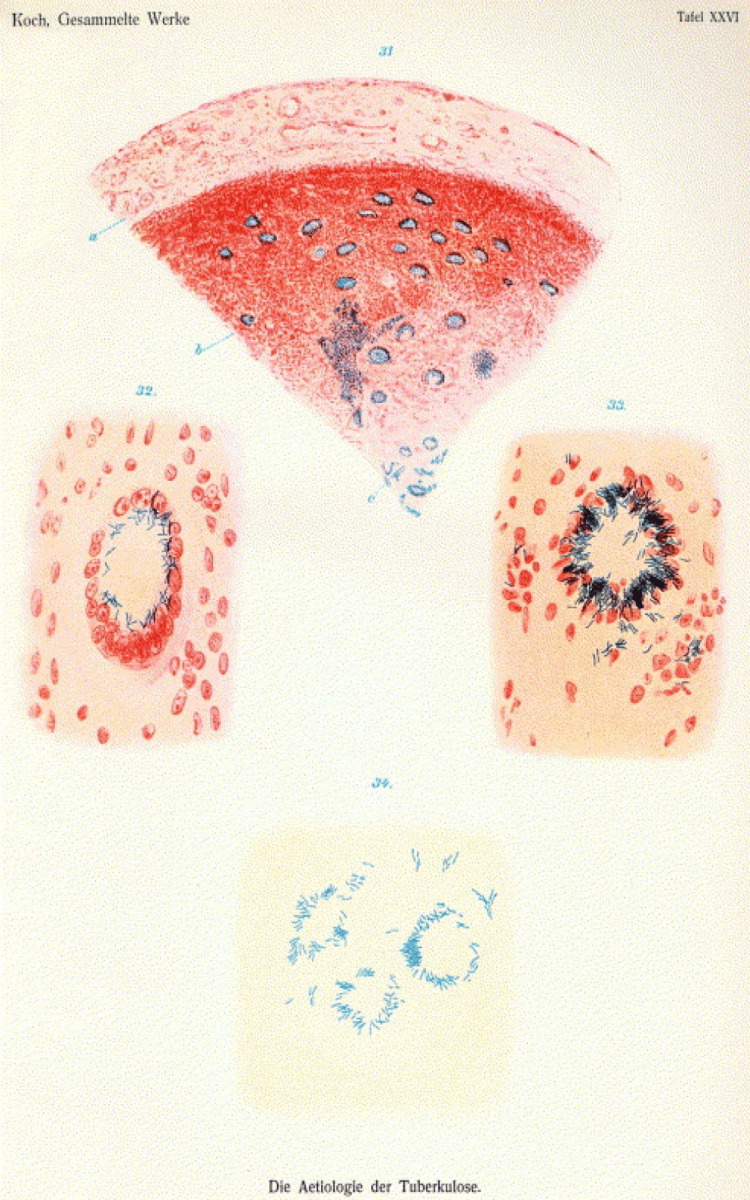
Illustrations from Robert Koch's "Die Ätiologie der Tuberkulose" (The Etiology of Tuberculosis), 1882, depicting the tubercle bacillus (Mycobacterium tuberculosis) in infected tissues, highlighting the groundbreaking work that identified the bacterium responsible for tuberculosis Source: Wikimedia commons/Public Domain

During his tenure as a government advisor for the Imperial Health Agency in Berlin during the 1880s, Robert Koch developed a keen interest in tuberculosis research. At the time, tuberculosis was widely believed to be hereditary, but Koch was convinced it was caused by a bacterium [13]. In 1882, he identified Mycobacterium tuberculosis as the causative agent of the disease and published his findings in "Die Ätiologie der Tuberkulose" ("The Etiology of Tuberculosis"). Koch presented his discovery to the German Physiological Society on March 24, 1882, noting that staining techniques revealed rod-shaped bacteria in tubercular tissue (Figure 3). Although initially met with skepticism from some scientists like Rudolf Virchow, Koch's findings were groundbreaking and praised by others, including Paul Ehrlich [14].

Koch's research on tuberculosis continued throughout his career (Table 1). In the mid-1880s, he focused on finding effective disinfection methods for tuberculosis sputum and experimented with arsenic and creosote, though these proved ineffective. He also tried to develop a tuberculosis vaccine, but his initial efforts were unsuccessful. By 1888, Koch shifted his focus to synthetic dyes for antibacterial testing. In 1890, Koch made a groundbreaking discovery: a glycerine-based extract from tuberculosis cultures exhibited the capacity to eradicate infected tissue in guinea pigs, thereby arresting the progression of the disease. He made a preliminary announcement at the Tenth International Medical Congress in Berlin and later demonstrated the extract's effectiveness in treating humans using the Bacillus Calmette-Guerin (BCG) technique [14].

**Table 1:** Koch's publications on tuberculosis Source: Sakula A: Robert Koch: centenary of the discovery of the tubercle bacillus, 1882

Year	Title	Publication	Notes/Significance
1882	Die Aetiologie der Tuberculose (The Etiology of Tuberculosis)	Berliner Klinische Wochenschrift 19: 221-30	Groundbreaking paper announcing the discovery of Mycobacterium tuberculosis, the bacterium that causes tuberculosis.
1883	Kritische Besprechungen der gegen die Bedeutung der Tuberkelbazillen gerichteten Publikationen (Critical Discussions on Publications Opposing the Role of Tubercle Bacilli)	Dtsch Med Wochenschr 9: 137-41	A critical review of publications that challenged the role of tubercle bacilli in tuberculosis.
1884	Die Aetiologie der Tuberculose (The Etiology of Tuberculosis)	Mittheilungen aus dem Kaiserlichen Gesundheitsamt 2: 1-88	Expanded and comprehensive investigation on the etiology of tuberculosis.
1890	Weitere Mittheilungen über ein Heilmittel gegen Tuberculose (Further Communications on a Cure for Tuberculosis)	Dtsch Med Wochenschr 16: 1029-32	Report on further developments regarding tuberculin, which Koch proposed as a treatment for tuberculosis.
1891	Fortsetzung der Mittheilungen über ein Heilmittel gegen Tuberculose (Continuation of Communications on a Cure for Tuberculosis)	Dtsch Med Wochenschr 17: 101-2	Continued discussion on the potential of tuberculin as a treatment for tuberculosis.
1891	Weitere Mittheilung über das Tuberkulin (Further Communication about Tuberculin)	Dtsch Med Wochenschr 17: 1189-92	Additional communications about the effectiveness and application of tuberculin.
1897	Die Bekämpfung der Tuberculose unter Berücksichtigung der Erfahrungen, welche bei der erfolgreichen Bekämpfung anderer Infektionskrankheiten gemacht sind (The Fight Against Tuberculosis Considering the Experiences Made in Successfully Combating Other Infectious Diseases)	Dtsch Med Wochenschr 27: 549-54	Presentation at the British Tuberculosis Congress discussing strategies for combating tuberculosis in light of experiences with other infectious diseases.
1901	Über die Agglutination der Tuberkelbazillen und über die Verwertung dieser Agglutination (On the Agglutination of Tubercle Bacilli and the Utilization of this Agglutination)	Dtsch Med Wochenschr 27: 829-34	Discussion on the agglutination of tubercle bacilli and the application of this process in diagnostics.
1905	Über die Immunisierung von Rindern gegen Tuberculose (with Schutz W., Neufeld F., Miessner H.) (On the Immunization of Cattle Against Tuberculosis)	Zeitschrift für Hygiene und Infektionskrankheiten 51: 300-27	Study on the immunization of cattle against tuberculosis.
1906	Über die Rolle der Milch bei der Übertragung der Tuberculose auf Menschen (On the Role of Milk in the Transmission of Tuberculosis to Humans)	Molkerei-Zeitung 16: 37	Examination of the role of milk in transmitting tuberculosis to humans.
1910	Epidemiologie der Tuberculose (Epidemiology of Tuberculosis)	Zeitschrift für Hygiene und Infektionskrankheiten 67: 1-48	Presentation at the Academy of Sciences in Berlin, covering the epidemiology of tuberculosis.

**Figure 4:**
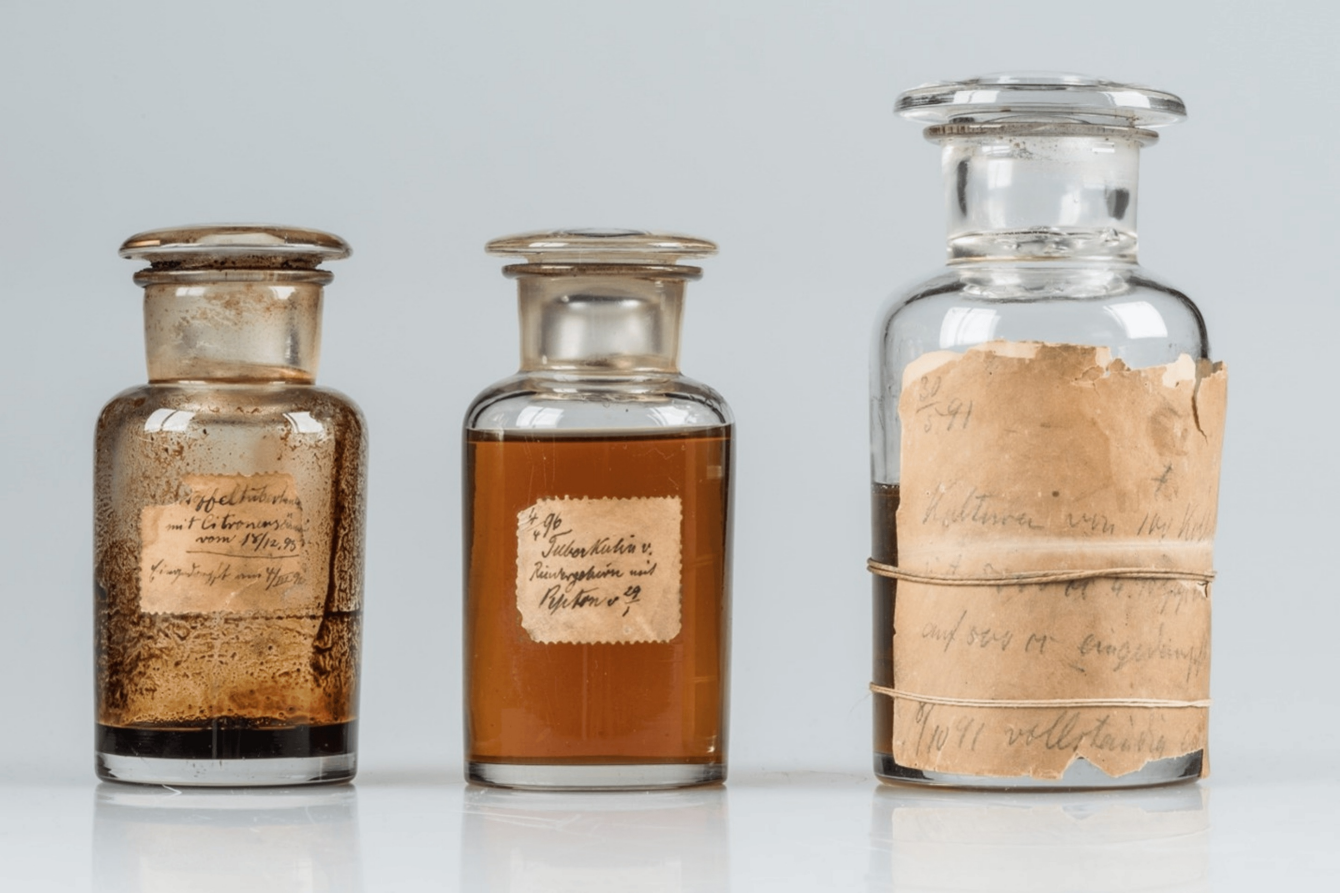
Tuberculin developed by Robert Koch as a cure for tuberculosis Source: Robert Koch Institute, Berlin Permission obtained from Robert Koch Institute.

Despite initial excitement, Koch's vaccine led to severe reactions in some test animals, known as Koch’s phenomenon. This reaction, marked by an extreme immune response at the vaccination site, reflected the complexity of tuberculosis treatment. Koch initially described the substance as a brownish, transparent fluid. The term "tuberculin" had been previously used by Josephs Pohl-Pincus in 1844 to refer to tuberculosis culture media, and Koch later adopted this terminology and referred to his extract as "tuberkulin" (Figure 4). Koch's work, published in early 1891, was initially seen as a breakthrough but is now regarded as a significant failure in its therapeutic application. While this endeavor severely damaged his reputation, he spent the remainder of his life relentlessly working to transform tuberculin into a viable medication. Despite this setback, Koch's discovery was not a complete failure, as the substance is now employed to assess tuberculosis-related hypersensitivity in patients [14].

Acquired Immunity

Koch's observations led to the understanding of acquired immunity. Subsequently, on December 26, 1900, he embarked on an expedition to German New Guinea. There, he studied the indigenous Papuan people and their blood samples, discovering the presence of Plasmodium parasites, which cause malaria. Despite this, the Papuans experienced only mild or even unnoticed malaria cases. In contrast, German settlers and Chinese laborers who had recently arrived fell ill with malaria more readily. Over time, however, these newcomers developed increasing resistance to the disease [15].

Koch's Postulates

While serving as a government advisor, Robert Koch detailed his discovery of the tuberculosis bacterium and its role in causing the disease. He emphasized the importance of isolating pathogens using pure cultures, methods summarized in Koch's postulates. These postulates, derived from his work on anthrax, established a foundational approach for identifying the causative agents of infectious diseases and became a benchmark in the field. Though Koch developed the principles, his assistant Friedrich Loeffler formalized them into postulates in 1883. The fourth postulate was later added by Erwin Frink Smith in 1905 [16].

1. The microorganism must be found in abundance in all individuals suffering from the disease, but should not be found in healthy individuals.

2. The microorganism must be isolated from a diseased individual and grown in pure culture.

3. The microorganism (from the pure culture) should cause disease when inoculated into a healthy, susceptible individual.

4. The microorganism must be re-isolated from the inoculated, diseased experimental host and identified as being identical to the original specific causative agent.

Sleeping Sickness

During the early 20th century, Robert Koch was tasked with studying tropical diseases, particularly trypanosomiasis, in German East Africa. However, his work in the region was not without controversy. While investigating these conditions, Koch made some advancements in understanding them. Nonetheless, his research methods in Africa raised significant ethical concerns. Koch's team conducted experiments on local populations without their informed consent or comprehension. In one prominent instance, they administered arsenic-based treatments to patients suffering from sleeping sickness, a therapy that proved highly toxic and frequently lethal. These actions have been criticized as unethical experimentation, reflecting the broader context of colonial exploitation and prevalent racism of the time period [17].

Awards and Honours

**Figure 5:**
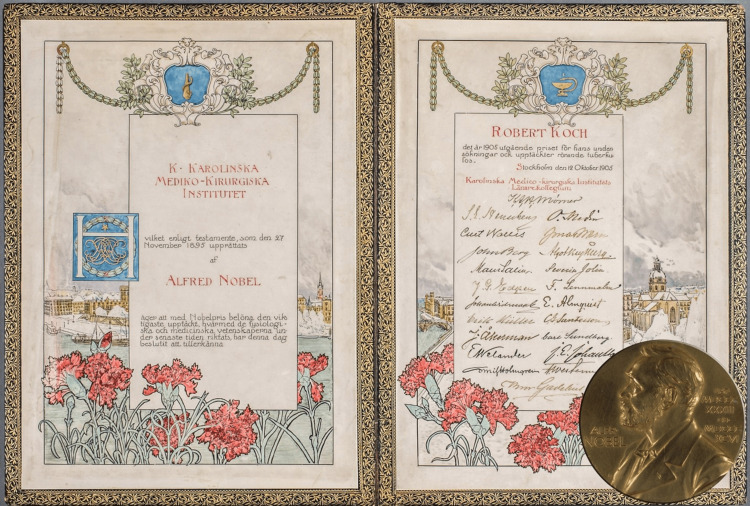
In 1905, Koch was awarded the Nobel Prize in Medicine for his discovery of the tuberculosis bacterium. Source: Archive of the Humboldt University of Berlin and Robert Koch Institute, Berlin Permission obtained from Robert Koch Institute.

Robert Koch’s exceptional contributions to the field of microbiology were widely recognized and rewarded. In recognition of his groundbreaking research on tuberculosis, he was elected as a Foreign Member of the Royal Society and received numerous honors, including the prestigious Knight Grand Cross of the Prussian Order of the Red Eagle. In 1905, he was awarded the Nobel Prize in Physiology or Medicine (Figure 5). In recognition of his continued contributions to tuberculosis and tropical disease research, he was further honored with the Order Pour le Mérite. The Robert Koch Medal was established to commend outstanding physicians to perpetuate his legacy. Moreover, the German Emperor conferred upon him the Order of the Crown, substantial financial rewards, and elevated his status to Privy Imperial Councillor and Surgeon-General of the Health Service. These accolades underscore Koch’s unparalleled impact on medical science [18].

**Figure 6:**
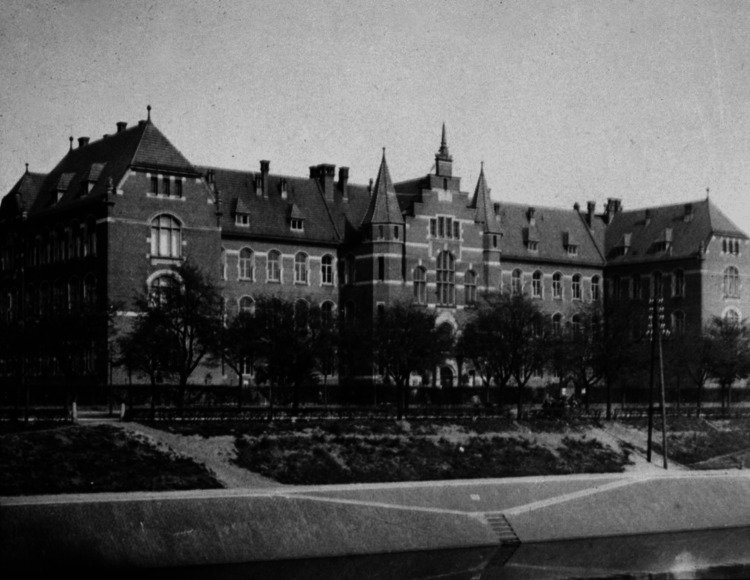
Royal Prussian Institute for Infectious Diseases in Berlin (1900) Source: Robert Koch Institute, Berlin Permission obtained from Robert Koch Institute.

**Figure 7:**
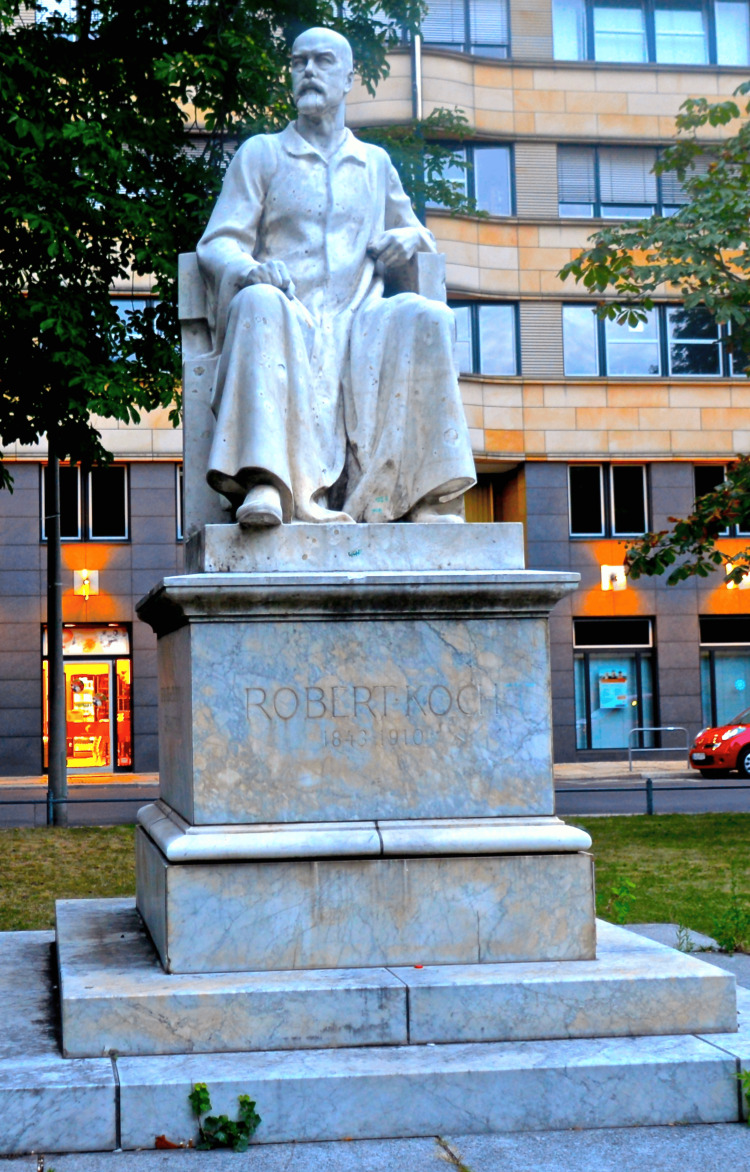
Statue of Koch at Robert-Koch-Platz in Berlin Source: Wikimedia Commons/Public Domain

Koch founded the Royal Prussian Institute for Infectious Diseases in Berlin in 1891, later renamed the Robert Koch Institute in recognition of his contributions (Figure 6). His enduring impact on global health is evidenced by the World Health Organization's designation of March 24 as World Tuberculosis Day, commemorating the anniversary of his seminal discovery of the tuberculosis bacterium. His legacy is also honored with a frieze at the London School of Hygiene & Tropical Medicine and a marble statue in Robert Koch Platz (Robert Koch Square) in Berlin (Figure 7). Koch’s life was depicted in a 1939 German film featuring Emil Jannings. Koch was also celebrated with a Google Doodle on his birthday in 2017. His relationship with Paul Ehrlich, who developed a tuberculosis diagnostic method, was portrayed in the 1940 film Dr. Ehrlich's Magic Bullet [18]. Ehrlich significantly enhanced the technique for staining mycobacteria, which earned him a commendation from Robert Koch. The innovative method introduced by Ehrlich would later serve as the foundation for the Ziehl-Neelsen acid-fast staining procedure, an approach that remains in use today [19].

## Conclusions

Robert Koch was a trailblazing German physician and microbiologist whose groundbreaking work laid the foundation for modern bacteriology. His identification of the pathogens causing tuberculosis, cholera, and anthrax advanced the germ theory of disease and significantly impacted public health. Koch's innovative techniques, including using agar for bacterial cultures and developing the Petri dish, revolutionized microbiological research. His postulates provided a rigorous method for linking specific microorganisms to diseases, cementing his role in medical science. Honored with numerous awards, including the Nobel Prize in 1905, Koch's legacy endures through World Tuberculosis Day and the Robert Koch Institute, celebrating his profound contributions to medicine.
